# Genetic basis of STEM occupational choice and regional economic performance: a UK biobank genome-wide association study

**DOI:** 10.1186/s40246-023-00488-2

**Published:** 2023-05-10

**Authors:** Chen Zhu, Qiran Zhao, Jianbo He, Petri Böckerman, Siyang Luo, Qihui Chen

**Affiliations:** 1 College of Economics and Management, China Agricultural University, No. 17 Qinghuadonglu, Haidian Dist., Beijing, China; 2 College of Agriculture, Nanjing Agricultural University, Nanjing, China; 3 School of Business and Economics, University of Jyväskylä, Jyvaskyla, Finland; 4 Department of Psychology, Sun Yat-Sen University, Guangzhou, China

**Keywords:** STEM, Occupational choice, Genome-wide association study, Polygenic score, Assortative mating, Comparative economic development

## Abstract

**Background:**

Science, technology, engineering, and mathematics (STEM) professionals are regarded as the highly skilled labor force that fosters economic productivity, enterprise innovation, and international competitiveness of a country. This study aims to understand the genetic predisposition to STEM occupations and investigate its associations with regional economic performance. We conducted a genome-wide association study on the occupational choice of STEM jobs based on a sample of 178,976 participants from the UK Biobank database.

**Results:**

We identified two genetic loci significantly associated with participants’ STEM job choices: rs10048736 on chromosome 2 and rs12903858 on chromosome 15. The SNP heritability of STEM occupations was estimated to be 4.2%. We also found phenotypic and genetic evidence of assortative mating in STEM occupations. At the local authority level, we found that the average polygenic score of STEM is significantly and robustly associated with several metrics of regional economic performance.

**Conclusions:**

The current study expands our knowledge of the genetic basis of occupational choice and potential regional disparities in socioeconomic developments.

**Supplementary Information:**

The online version contains supplementary material available at 10.1186/s40246-023-00488-2.

## Introduction

STEM (Science, technology, engineering, and mathematics) jobs involve the use of technical knowledge and skills to solve daily and societal problems [5]. It is widely agreed that STEM professionals make up the crucial part of the workforce that sustains a country’s economic growth, enterprise innovation, and global competitiveness [9]. However, in the global labor market, several countries, such as the USA and the UK, are increasingly facing a shortage of STEM-related professionals, especially in the government sector and private industry [17, 23]. The emergence of trends showing a dearth of individuals choosing careers in STEM fields has garnered significant attention from both the academic community and the public alike, spurring efforts to reform and improve STEM education. Recent research has investigated various individual and environmental factors that may influence an individual's career path, including personality traits, mathematical ability, motivation, childhood exposure, and ability beliefs, as well as parental occupation, peer effects, and gender stereotypes [4, 12, 21]. Despite this progress, little is currently known about the potential role of genetic predispositions in shaping occupational choices, including the likelihood of pursuing a career in STEM.

On the other hand, recent studies have convincingly demonstrated the heritability of non-STEM occupations, with Nicolaou and Shane's [15] twin-based research revealing high heritability in professions such as teaching, management, sales, and self-employment. The findings of Roeling et al. [18] estimated the heritability of creative professions to be approximately 0.70, and more recently, Song et al. [19] discovered nine genetic loci that were significantly linked to leadership roles using a large-scale genome-wide association study. Despite these compelling results, it is noteworthy that no specific genetic variants have yet been empirically linked to occupational choices in STEM fields, to the best of our knowledge.

The principal objective of this study is twofold. First, we aim to advance our knowledge of the genetic predisposition engaging in occupational choice at the individual level, by conducting a genome-wide association study of STEM occupations using the large-scale UK Biobank (UKB) database. We adopted the general definition used in labor economics studies and measured the STEM phenotype based on the UK Standard Occupational Classification (SOC) 2000 system. We also calculated the heritability of STEM occupations and tested for assortative mating based on the main GWAS results. Second, we investigated the genetic associations between STEM occupations and a number of regional economic performance metrics. This allows us to explore the potential genetic basis of regional socioeconomic disparities discussed in the literature on comparative economic development [3].

## Methods

### Measures of STEM occupations in UKB

Our analysis draws on a unique dataset from the UKB. UKB is a population-based sample that comprises over 500 thousand UK residents recruited since 2006. During a verbal interview conducted by trained staff, participants were asked about their job status and job title categorized by the SOC 2000 system. We used these questions to construct a phenotypic measure of STEM occupations. In our analysis, all UKB participants with occupations within the sub-major category of “Science and Technology Professionals” were classified as having a STEM job, and we coded these participants as STEM professionals (1) and others as non-STEM professionals (0). This occupation sub-category contains a broad range of science, engineering, information, and communication technology professionals. A detailed list of occupations included in the case-cohort is documented in Additional file 1: Table S1. Participants with missing SOC 2000 data were excluded from the present study (Additional file 1: Fig. S1).

### Genotyping and imputation

Genotypes of the UKB data set were analyzed with the Affymetrix (Santa Clara, CA, USA) UKB Axiom Array and the UK BiLEVE Axiom Array. Further information about quality control procedures, imputation, and principal components analysis can be found in Bycroft et al. [7]. Participants were removed from the present study based on the following characteristics: non-British ancestry, high missingness, relatedness, quality control failure, and gender mismatch.

### Statistical analyses

We performed GWAS using the machine-learning-based method REGENIE [13] of the STEM trait across 9273 cases and 169,703 controls of European descent from the UKB data. REGENIE is an efficient two-step whole-genome regression method for genetic association tests that accounts for both sample relatedness and population structure. Firth logistic regression test was used for the binary trait of STEM jobs. The REGENIE method involves two steps. In step 1, we included directly genotyped variants (a total of 588,699) with a minor allele frequency (MAF) greater than 1%, missingness lower than 10%, and Hardy–Weinberg equilibrium test *p*-value greater than 1 × 10^–15^. In step 2, we performed the single-variant association testing using the imputed dataset (a total of 7,677,418 autosomal variants) with covariates of age, sex, and the first ten ancestral principal components. Independent significant loci were identified as those with *p* < 5 × 10^–8^, *r*^*2*^ < 0.1, and distance > 250 kb. Based on effect estimates from GWAS results, we built polygenic scores of STEM for variants meeting the *p*-value threshold of 0.05 using PLINK. We calculated each PGS as the sum of imputed allele *j* dosages carried by a respondent *i* (*SNP*_*j,i*_) multiplied by the estimated effect size (*β*_*j*_), i.e., ${\text{PGS}}_{i} = \sum\nolimits_{j = 1}^{J} {\beta_{j} {\text{SNP}}_{j,i} }$, and normalized the PGS between 0 and 1. We then used linkage disequilibrium score regression (LDSC; [6] to examine genetic correlations across the STEM career choice and a range of behavior and physiological traits. Finally, we conducted a phenome-wide association study (PheWAS) to identify phenotypes that lead SNPs were associated with from previous GWAS work using the GWAS Atlas [22].

## Results

### GWAS for STEM occupations

As reported in Table 1, the data involve 178,876 participants of European ancestry that passed quality control from the UKB database, among whom 9273 (5.2%) had a STEM career. In the pooled sample (*N* = 178,876), 47.8% were males, 34.9% held a college or university degree, and the average age was 54.3 (s.d. = 7.6). The share of male participants among STEM professionals (*N* = 9273) was 83.5%, which is much higher than that among non-STEM workers (45.9%), suggesting a notable gender gap in the professional STEM field in the UK labor market.

**Table 1:** Summary statistics of the analytical sample in UKB

Variable	(1) Pooled		(2) STEM participants		(3) Non-STEM participants	
	Mean	SD	Mean	SD	Mean	SD
Age	54.3	7.6	53.4	7.9	54.4	7.6
Male	47.8%	–	83.5%	–	45.9%	–
College/university degree	34.9%	–	56.9%	–	33.8%	–
STEM occupation	5.2%	–	100.0%	–	0.0%	–
Height (centimeters)	170.8	9.2	175.4	8.1	170.5	9.2
Weight (kilograms)	78.6	15.9	82.9	14.7	78.4	16.0
BMI (kg/m^2^)	27.3	4.7	27.0	4.2	27.3	4.7
Household size	2.6	1.3	2.6	1.3	2.6	1.3
Household income (pounds)	65,593.9	46,458.2	78,087.8	44,435.4	64,915.4	46,469.5
N	178,976		9273		169,703	

**Fig. 1:**
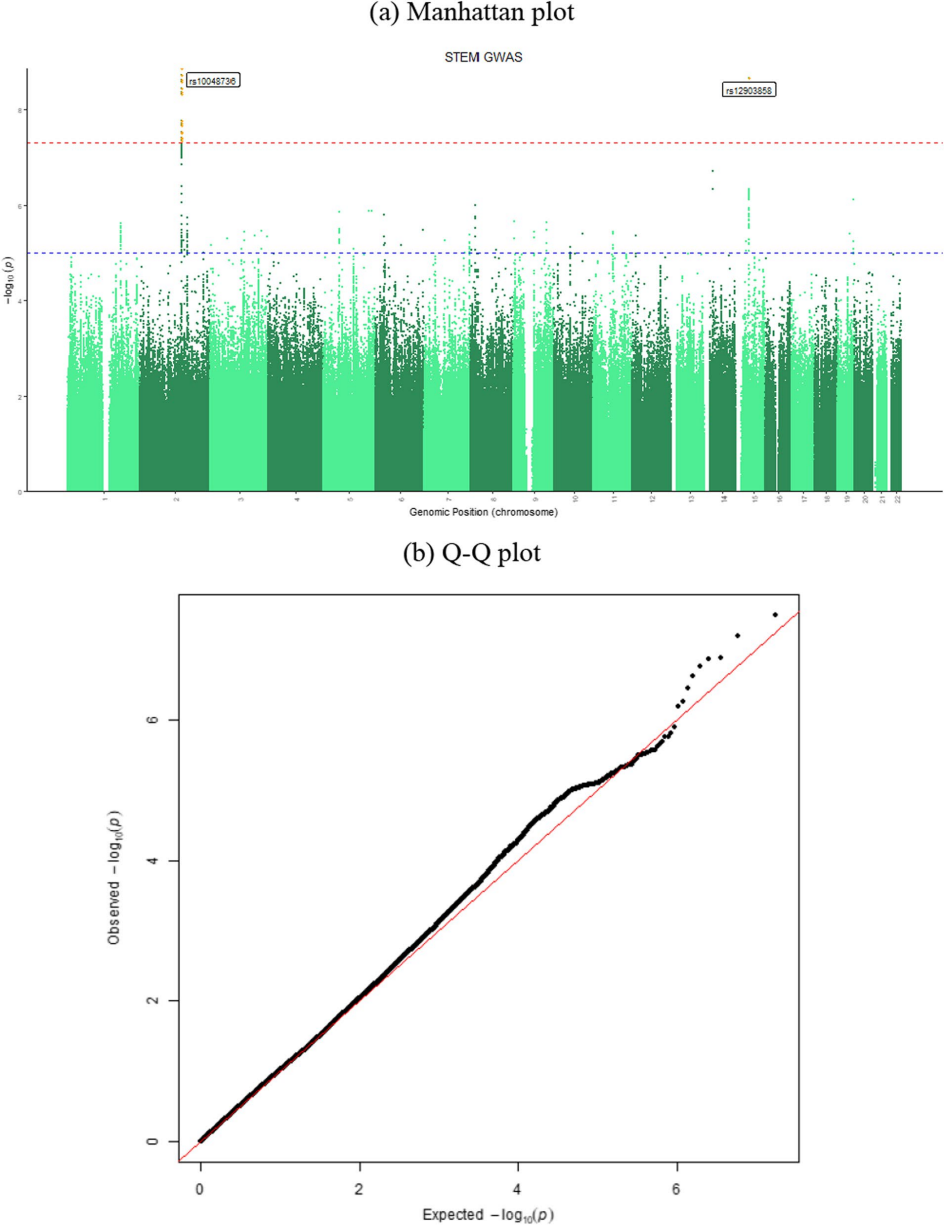

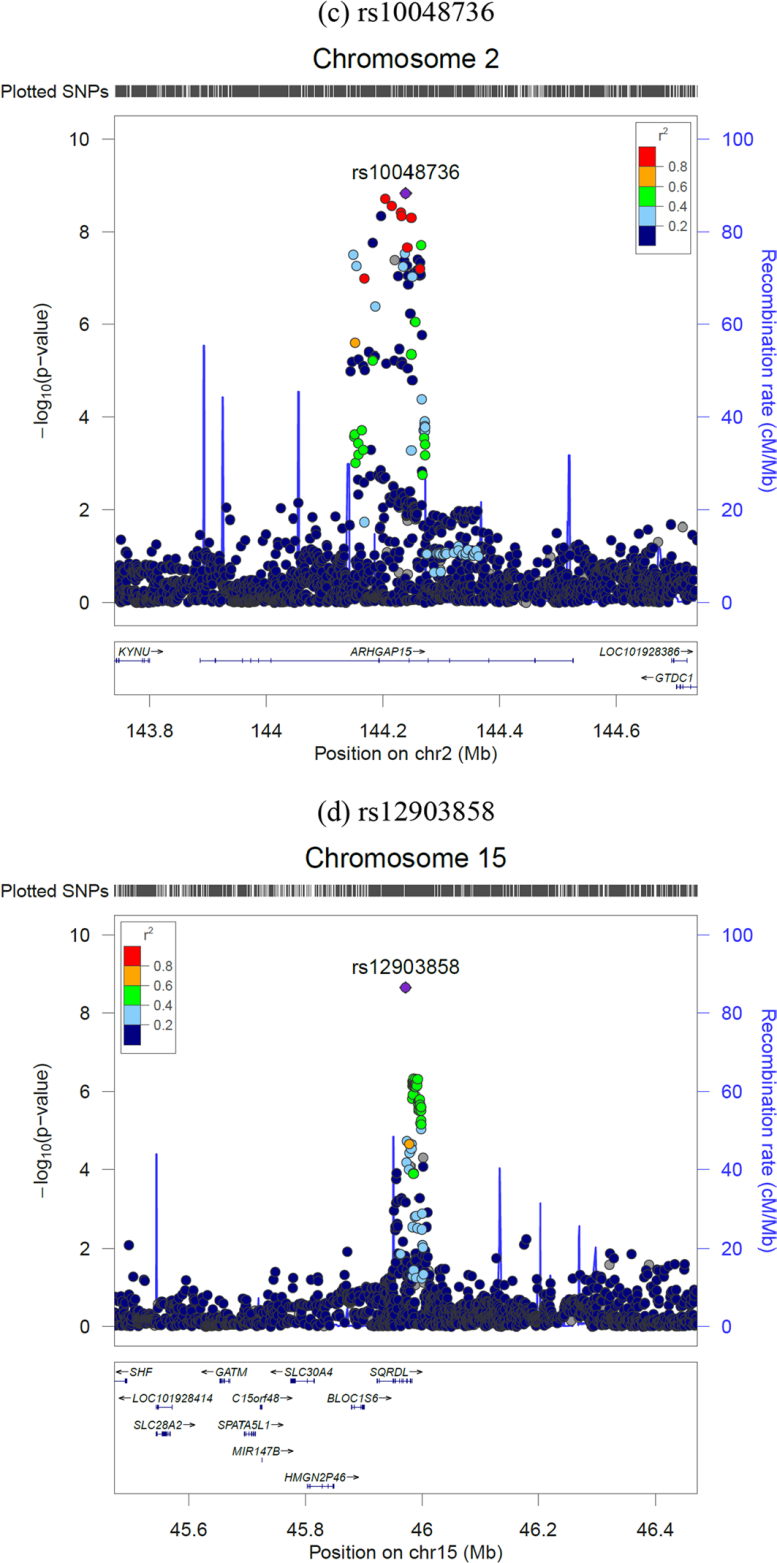
Manhattan plot of the career choice of STEM and LD zoom plots of lead SNPs

We identified two genome-wide significant (*p* < 5e−8) single-nucleotide polymorphisms for the binary trait of STEM (Fig. 1 and Table 2): rs10048736 on chromosome 2 and rs12903858 on chromosome 15. Using LocusZoom [16], SNPs within 500 kb of the lead SNPs are plotted in Fig. 1. The genomic inflation factors ($\lambda_{GC}$) was estimated to be 1.07, suggesting no substantial impact of population structure or unmodeled relatedness. The NHGRI-EBI GWAS Catalog showed links between lead SNPs and genes with neuroticism, educational attainment, body mass index, sleep-related phenotypes, corpus callosum mid-posterior volume, and Parkinson’s disease progression.

**Table 2:** Summary of top loci for STEM trait identified from GWAS

SNP	CHR	BP	A1	A2	EAF	Beta	SE	P	Nearest Gene(s)	Function
rs10048736	**2**	**144,239,303**	**A**	**G**	**0.36**	**0.086**	**0.014**	**1.46E**−**09**	***ARHGAP15***	**Intron Variant**
rs12691680	2	144,204,338	T	C	0.36	0.086	0.014	1.94E−09	*ARHGAP15*	Intron Variant
rs12903858	**15**	**45,972,166**	**C**	**T**	**0.43**	**− 0.086**	**0.014**	**2.26E**−**09**	***SQOR/SQRDL***	**Intron Variant**
rs770075436	2	144,228,576	T	TAAAG	0.35	0.085	0.014	2.45E−09	*ARHGAP15*	Intron Variant
rs13411140	2	144,215,811	T	C	0.36	0.085	0.014	2.74E−09	*ARHGAP15*	Intron Variant
rs28684621	2	144,231,584	G	T	0.36	0.084	0.014	3.77E−09	*ARHGAP15*	Intron Variant
rs56081031	2	144,197,423	C	A	0.27	− 0.092	0.016	4.56E−09	*ARHGAP15*	Intron Variant
rs28380327	2	144,232,491	T	A	0.36	0.084	0.014	4.59E−09	*ARHGAP15*	Intron Variant
rs35789697	2	144,248,718	A	G	0.35	0.084	0.014	4.89E−09	*ARHGAP15*	Intron Variant
rs2381455	2	144,182,917	G	T	0.27	− 0.089	0.016	1.73E−08	*ARHGAP15*	Intron Variant
rs6705184	2	144,265,362	G	C	0.5	0.079	0.014	1.96E−08	*ARHGAP15*	Intron Variant
rs4662334	2	144,241,887	G	A	0.36	0.08	0.014	2.20E−08	*ARHGAP15*	Intron Variant
rs12617059	2	144,238,363	T	G	0.27	− 0.087	0.016	2.91E−08	*ARHGAP15*	Intron Variant
rs12999615	2	144,149,614	A	T	0.27	− 0.087	0.016	3.08E−08	*ARHGAP15*	Intron Variant
rs4402695	2	144,259,542	T	C	0.27	− 0.087	0.016	3.94E−08	*ARHGAP15*	Intron Variant
rs57014442	2	144,220,812	AAT	A	0.27	− 0.086	0.016	4.04E−08	*ARHGAP15*	Intron Variant
rs35564832	2	144,237,261	G	A	0.27	− 0.086	0.016	4.14E−08	*ARHGAP15*	Intron Variant
rs13020925	2	144,263,416	C	T	0.27	− 0.087	0.016	4.52E−08	*ARHGAP15*	Intron Variant

The bold SNPs are independent genetic loci associated with participants’ choice of STEM jobs

### Heritability and genetic correlations of STEM occupations

For the trait of STEM occupation, the LDSC SNP-based heritability (*h*^*2*^) was estimated to be 4.2% (95% CI, 2.8–5.6%). The SNP heritability of STEM occupation is smaller than physical traits but generally in accordance with some of the behavioral traits, such as risk tolerance (*h*^*2*^ = 4.6%; [11] and leadership position (*h*^*2*^ ranged from 3 to 8%; [19]

**Fig. 2:**
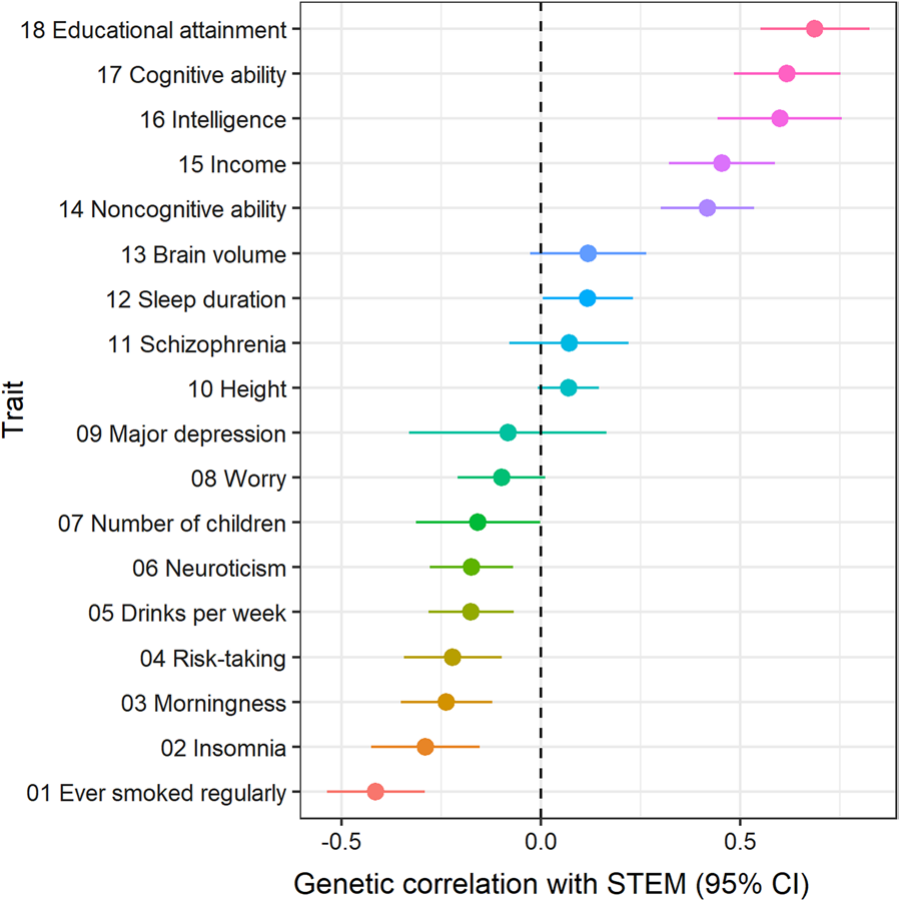
Genetic correlations of STEM occupation phenotype with a variety of health/behavior traits

Next, we examined genetic correlations between STEM occupations with 17 potentially linked personal traits using GWAS summary statistics from previous studies, including educational attainment, intelligence, personality, risk preference, height, brain volume, income, and sleep duration. As shown in Fig. 2, we found significant positive genetic correlations of STEM occupation with educational attainment (*r*_g_ = 0.68, 95% CI 0.55–0.82), cognitive ability (*r*_g_ = 0.62, 95% CI 0.48–0.75), intelligence (*r*_g_ = 0.60, 95% CI 0.44–0.75), household income (*r*_g_ = 0.45, 95% CI 0.32–0.59), noncognitive ability (*r*_g_ = 0.42, 95% CI 0.30–0.53), and sleep duration (*r*_g_ = 0.12, 95% CI 0.00–0.23); and significant negative genetic correlations with ever smoked regularly (*r*_g_ = − 0.41, 95% CI − 0.53 to − 0.29), insomnia (*r*_g_ = − 0.29, 95% CI − 0.43 to − 0.15), morningness (*r*_g_ = − 0.24, 95% CI − 0.35 to − 0.12), risk-taking (*r*_g_ = − 0.22, 95% CI − 0.34 to − 0.10), drinks per week (*r*_g_ = − 0.18, 95% CI − 0.28 to − 0.07), neuroticism (*r*_g_ = − 0.17, 95% CI − 0.28 to − 0.07), and number of children (*r*_g_ = − 0.16, 95% CI − 0.31 to − 0.00).

### The explanatory power of the STEM polygenic score

To examine the explanatory power of the polygenic score (PGS) of STEM, we constructed the PGS based on the GWAS results (*p*-value threshold of 0.05; standardized between 0 and 1) and obtained parameters from multiple regression analyses of various socioeconomic outcomes, including STEM jobs, educational attainment (measured by whether a participant holds a college/university degree), and household income (Table 3).^1^ All regressions were adjusted for age, age-squared, sex (except for rows *b* and *c*), educational attainment (except for column 2), and the first ten genetic principal components (PCs) of each participant.

**Table 3:** Parameter estimates of STEM polygenic score in explaining socioeconomic attainment

UKB Cohort	(1) STEM jobs			(2) Educational attainment			(3) Income		
	Beta	*p*	*N*	Beta	*p*	*N*	Beta	*p*	*N*
(a) Pooled	0.764	< 0.001	315,400	0.455	< 0.001	487,202	6340.032	< 0.001	415,766
(b) Males	1.169	< 0.001	150,713	0.532	< 0.001	223,043	5395.146	< 0.001	198,227
(c) Females	0.318	< 0.001	164,687	0.379	< 0.001	264,159	6744.243	< 0.001	217,539
(d) Irish	0.667	< 0.001	8617	0.395	< 0.001	12,707	5826.574	0.246	11,133
(e) Indian	0.990	< 0.001	4092	0.459	< 0.001	5660	13,444.807	0.150	4253
(f) Caribbean	0.522	< 0.001	3217	0.185	0.081	4295	21,886.042	0.011	3305
(g) African	1.399	< 0.001	2212	0.417	0.009	3202	24,189.643	0.038	2445
(h) Chinese	1.629	< 0.001	1112	0.218	0.264	1502	615.138	0.977	1201

All regressions were adjusted by age, age-squared, sex (except for rows *b* and *c*), and the first ten genetic principal components of each participant. Regressions of STEM jobs and income were also adjusted by whether holding a college/university degree

In the pooled UKB cohort (Table 3, row *a*), a 0.1 increase in the STEM polygenic score was associated with a 7.6% increase in the probability of being a STEM professional, a 4.6% increase in the probability of earning a college/university degree, and 634-pound increase in annual household income. Moreover, for the phenotype of STEM jobs, the STEM polygenic score accounted for 3.5% of the variance. This is on top of sex and educational attainment, which accounted for 0.1% and 1.4% of the variance of STEM jobs, respectively. Interestingly, the estimated effects of PGS on the propensities to have a STEM job and a college/university degree were found to be more pronounced in males than in females, but the opposite is found for household income (Table 3, rows *b* and *c*).

We also estimated the effects of STEM PGS in five independent UKB cohorts with ethnic backgrounds different from the original discovery dataset (i.e., White British), including Irish (*N* = 13,108), Indian (*N* = 5835), Caribbean (*N* = 4420), African (*N* = 3308), and Chinese (*N* = 1538). As shown in Table 3, rows *d* to *h*, the STEM polygenic score is significantly associated with the phenotype of STEM jobs in all five independent UKB subsamples (*p* < 0.0001). For educational attainment, the PGS of STEM was found to be a significant predictor in Irish (*p* < 0.0001), Indian (*p* < 0.0001), and African (*p* = 0.009) subsamples. For household income, the STEM PGS was found to be a significant predictor only in the Caribbean (*p* = 0.011) and African (*p* = 0.038) subsamples.

### Assortative mating and intergenerational occupational transmission of STEM occupations

To test for assortative mating of STEM jobs at both genetic and phenotypic levels, we identified 39,985 couples as pairs of unrelated (i.e., genetic relationship < 0.05) opposite-sex individuals matched on several household variables as described previously [8, 24]. We found significant correlations between couples for STEM occupations both genetically (*ρ* = 4.3%, *p* < 0.0001) and phenotypically (*ρ* = 11.0%, *p* < 0.0001), lending support to the existence of assortative mating and economic homogamy from the perspective of occupational choices [10, 24].

We also identified 3708 parent–offspring pairs based on the kinship coefficients described in Cheeseman et al. [8] to examine the intergenerational transmission of STEM occupations. Surprisingly, although we found a significant genetic correlation of STEM jobs between parents and offspring (*ρ* = 52.9%, *p* < 0.0001), the phenotypic correlation of actual STEM occupational choices is insignificant (*ρ* = 4.5%, *p* = 0.21), implying a potential higher intergenerational occupational mobility and more employment opportunities for offspring of STEM parents [14].

### Associations between average STEM polygenic score and regional economic performance

**Fig. 3:**
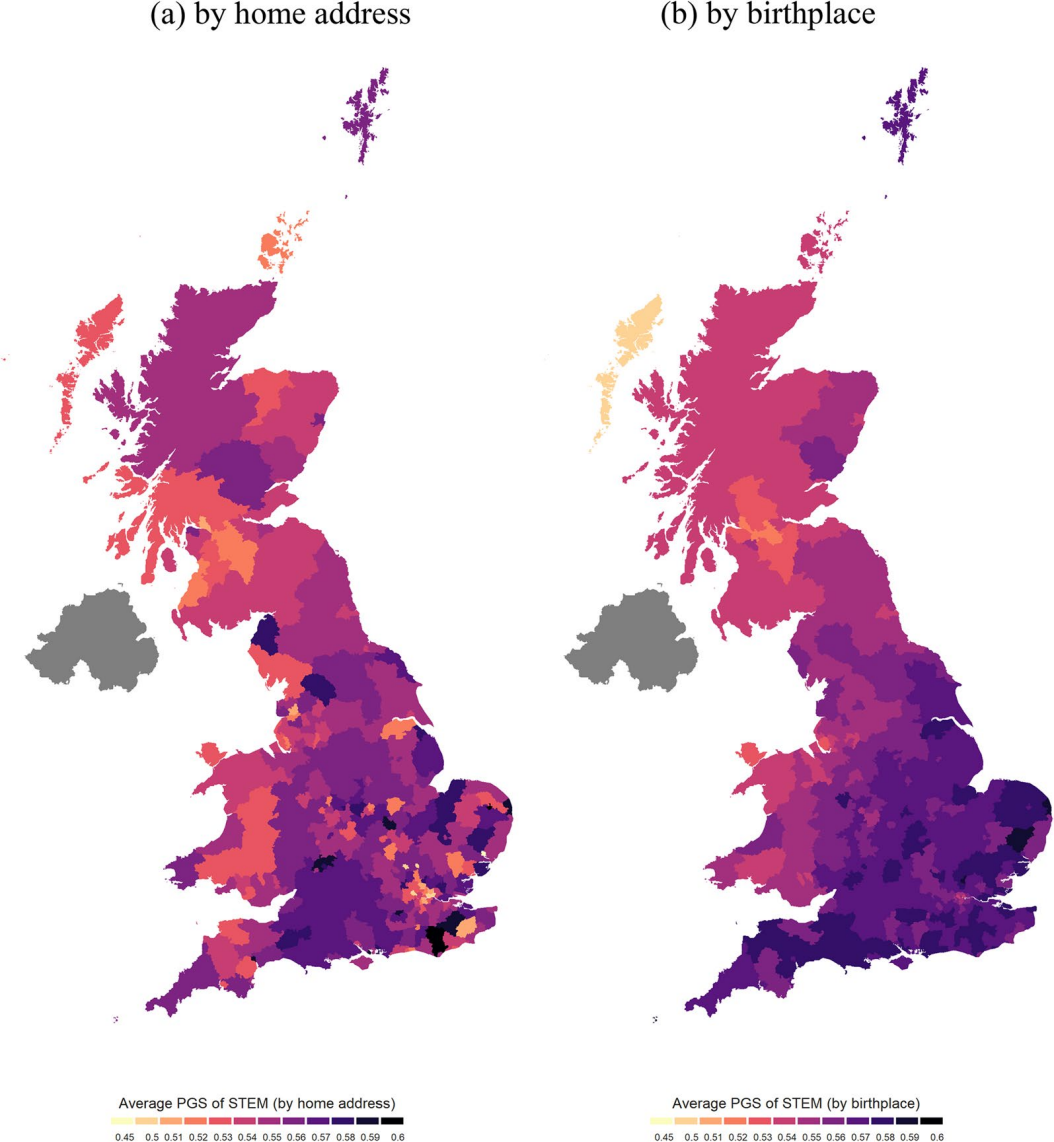
Geographic distributions of STEM polygenic scores by home address and birthplace in UKB. *Notes*: Color bars indicate the regional average STEM polygenic score

Finally, we investigated whether the regional average STEM polygenic score is associated with the economic performance of local administrative authorities in the UK. Figure 3 shows the geographic distributions of the average STEM polygenic score at the local authority level based on both current home address (a) and birthplace (b) provided in the UKB. We tested the associations between the average STEM polygenic score (based on home address) and four publicly available indicators of regional economic performance, including gross domestic product (GDP) from 1998 to 2019, value-added tax (VAT) from 1998 to 2019, counts of business by industry groups in 2016, and employments of business by 17 industry groups in 2016 of 380 local administrative units in UKB dataset. All regional data were collected from the UK’s Office for National Statistics (ONS) website.

**Fig. 4:**
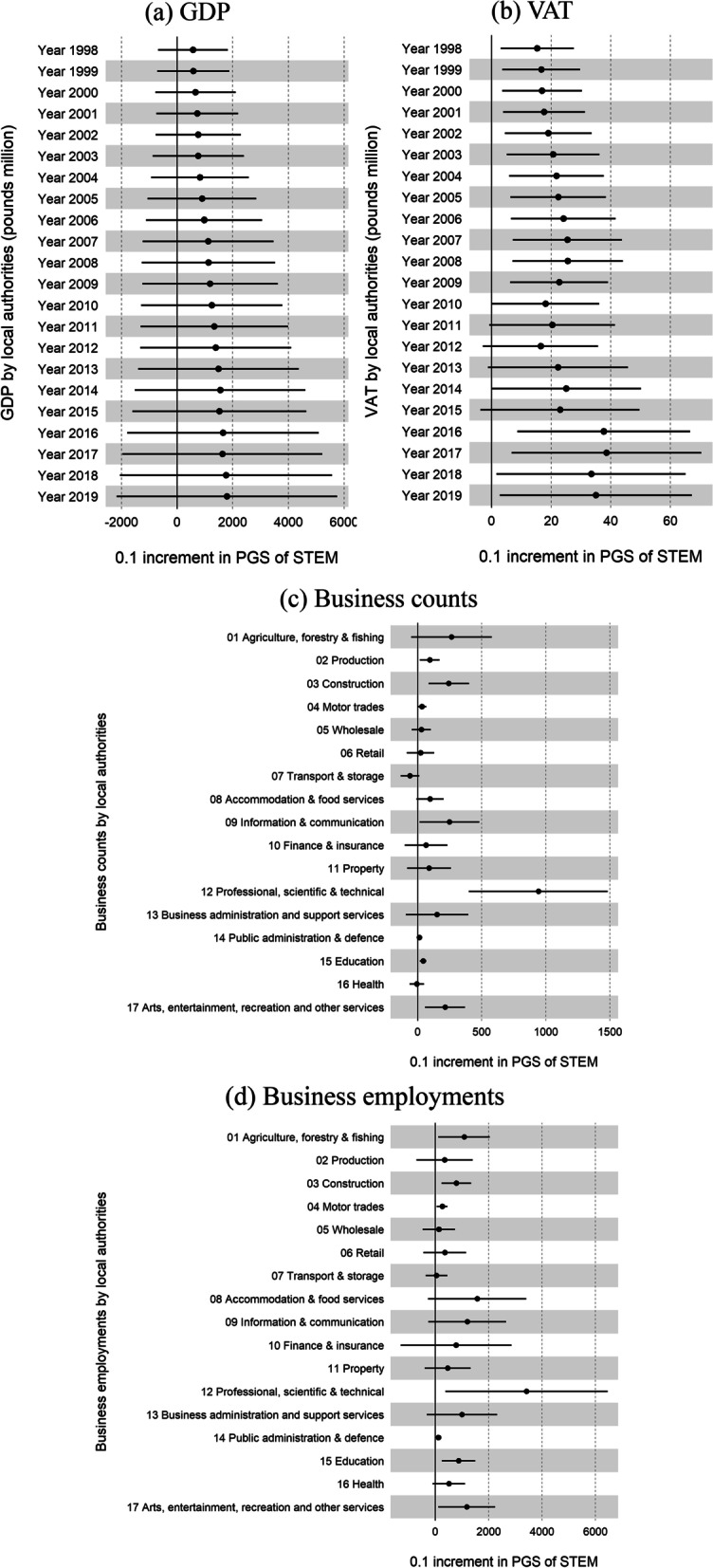
Associations between an increase of 0.1 in average STEM polygenic score and regional economic performance by local authorities based on home address

Our empirical specifications include a full set of regional fixed effects at the upper regional level (i.e., East Midlands, East of England, London, North East, North West, Scotland, South East, South West, Wales, West Midlands, and Yorkshire and The Humber) that account for all permanent differences such as time-invariant environmental effects between regions over time. Moreover, we control for population at the local authority level. As shown in Fig. 4, after controlling for local population and regional fixed effects, the STEM PGS has a statistically significant association with VAT (panel *b*; 1998–2009, and 2016–2019), but not with GDP (panel *a*). In terms of local business counts, a 0.1 increase in the average STEM polygenic score is significantly associated with 942 more business counts in professional, scientific & technical, 250 more in information & communication, 245 more in construction, 215 more in arts, entertainment, recreation and other services, 96 more in production, 44 more business counts in education, 35 more in motor trades, and 16 more in public administration & defence. Similarly, for local employments of business, a 0.1 increase in the average STEM polygenic score is significantly associated with 3415 more employment in professional, scientific & technical, 1176 more in arts, entertainment, recreation and other services, 1079 more in agriculture, forestry & fishing, 868 more in education, 783 more in construction, 256 more in motor trades, and 113 more in public administration & defence.^2^

VAT is a consumption tax that is paid by the final consumer and collected by businesses on behalf of the government. It has been linked to economic efficiency [2]. On the other hand, GDP is a measure of the economic output of a country. Regional business counts and employment are crucial indicators of the local market and business performance. In line with findings from Abdellaoui et al. [1], the results presented here suggest that some of the regional economic outcomes, such as VAT and local business performance, are directionally linked to STEM-associated alleles detected by GWAS conducted in this study. However, it is important to interpret these links with caution, as they may not necessarily be causal. Other factors such as labor migration and business reallocation might also be driving these links.

## Conclusion and discussion

Against the background of an increasing shortage of STEM-related professionals in several countries such as the USA and the UK, the role of STEM education and jobs in revitalizing economic growth and the labor market in these countries has been subject to heated debate [17, 23]. The present study helps to inform this debate by empirically linking individuals’ STEM-related job choices with their genetic markups and estimating the associations between STEM polygenic scores and the indicators of individual socioeconomic attainment and regional economic prosperity. Probing into these previously unexplored topics based on a large-scale dataset provided in the UKB, our GWAS also contributes to the fields of education, labor, and regional economics.

Our study first identified two independent genetic loci associated with participants’ choice of STEM jobs (the most significant SNP in each of the two loci being rs10048736 on chromosome 2 and rs12903858 on chromosome 15). Further estimations revealed strong positive associations between individuals’ STEM PGS and their socioeconomic attainment (measured by college attendance and household income). We also demonstrated statistically significant associations between the average STEM PGS and several indicators of regional economic performance (including local VAT, business counts, and business employment). Pieced together, these findings suggest that driven by STEM-related genetic markups, individuals choosing a STEM-related career may perform better in the labor market (through completing more years of STEM education), which may in turn improve local economic performance.

While most of our findings point in the same direction, important heterogeneity remains. First, there is a significant gender gap in the STEM PGS-socioeconomic attainment association. More specifically, the estimated associations between the STEM PGS and the probabilities of having a STEM job and a college/university degree are much larger in males than in females. This difference might reflect the influence of environmental factors (such as gender stereotyping) that suppress the effect of STEM PGS among females. Interestingly, the STEM PGS-household income association is more pronounced in females, which might reflect factors in the marriage market that we were unable to account for in our analysis.

Secondly, there are also differences in the STEM PGS-socioeconomic attainment association across ethnic subgroups. Take the Chinese subgroup, for example. While the STEM PGS-STEM job linkage among Chinese is highest among all five ethnic subgroups examined, the STEM PGS-college degree and STEM PGS-income linkages (—the latter is statistically significant) among Chinese are nearly the lowest among the five. These ethnic disparities might be due to cultural factors. For example, while the genetic makeup induces Chinese individuals to pursue STEM education, the possible lack of entrepreneurship in the Chinese culture may dampen the effect of STEM PGS on their income. A potentially segmented labor market in the UK could also be a factor, since the segmentation might be due to demographic, social, cultural and political reasons. These ethnic disparities suggest that while promoting STEM education may be able to revitalize economic progress and promote individual welfare in general, policies tailored to different subgroups’ cultural and ethnic backgrounds may be needed to fully realize the potential of STEM education and STEM jobs.

The findings of this study have several potential applications. Firstly, this study provides valuable information for policymakers and educators who are interested in promoting STEM education and jobs as a means of revitalizing local economic growth and improving individual socioeconomic outcomes. Secondly, this study highlights the importance of considering individual genetic makeup in understanding and addressing socioeconomic disparities. Thirdly, this study contributes to the growing body of the literature on the genetics of complex traits and behaviors. Our findings also have implications for social equality. Specifically, we found significant differences in the associations between the STEM polygenic score and socioeconomic outcomes across gender and ethnic subgroups. These findings suggest that policies tailored to different subgroups’ cultural and ethnic backgrounds may be needed to fully realize the potential of STEM education and STEM jobs and promote social equality. For example, interventions that target gender stereotyping in education and the workplace may help to reduce the gender gap in the associations between STEM polygenic scores and socioeconomic outcomes. Similarly, interventions that promote entrepreneurship and innovation in ethnic subgroups where these factors are lacking may help to enhance the effect of STEM polygenic scores on income and promote social equality.

Before closing, a note on the limitations of this study is in order. First, despite the wealth of information provided in the UKB database, our study still lacks sufficient controls for environmental factors. Thus, the associations between STEM PGS and economic indicators we found might not necessarily reflect causation; environmental factors might moderate this association. Second, data limitations may have also prevented us from depicting some of the associations of interest. For example, the UKB contains data only on household income rather than wage or labor income at the individual level. As such, household composition and factors from the marriage market may affect the estimated effect of STEM PGS on individuals’ labor productivity. Thirdly, we cannot account for potential time-variant regional effects in our empirical models of the regional economic analyses. Finally, as with other GWAS, a fundamental methodological challenge is that population stratification can occur even in homogeneous populations [20]. In that case, the associations we found might have been driven by demographic changes rather than genetic factors.

Despite these limitations, we believe that our analysis has provided novel and valuable information to advance knowledge about genetic predisposition engaging in STEM job choices. We also hope our study can attract more GWAS or related studies on the nexus of genetic disposition, educational attainment, and labor market performance.

## Supplementary Information

**Additional file 1.** Supplemental tables and figures.

## Data Availability

The data that support the findings of this study are available from UK Biobank but restrictions apply to the availability of these data, which were used under license for the current study, and so are not publicly available. Data are however available from the corresponding authors upon reasonable request and with permission from UK Biobank. The GWAS summary statistics for STEM occupational choice can be downloaded from GWAS Catalog soon.

## Footnotes

1 Specifically, for binary outcomes of STEM jobs and earning a college/university degree, linear probability models were used. for continuous outcome of household income, a multiple linear regression model was used.

2 We also tested the association between the average STEM PGS and local economic performance based on birth place. Results (Additional file 1: Fig. S2) indicate that the regional average STEM PGS is also associated with the economic performance of local administrative authorities by birthplace, but the associations are economically smaller and less significant. We believe that this difference could be due to the internal/domestic migration that occurs within the UK. In our sample, 67.7% participants had relocated to a different local authority from birthplace. When people move to a new location, they tend to work and consume more in that place, rather than in the place where they were born. Therefore, the associations between the average STEM PGS and local economic performance are more pronounced based on home address, rather than birthplace.
