# Supplementary material for: Genetic basis of STEM occupational choice and regional economic performance: a UK biobank genome-wide association study
Source: Hum Genomics. 2023 May 10;17:40. doi: 10.1186/s40246-023-00488-2 (PMC10170832; doi:10.1186/s40246-023-00488-2)

**Supplementary Table 1**. List of STEM occupations

| SOC2000 | STEM Occupation |
| --- | --- |
| 21110 | Chemists |
| 21111 | Research/Development Chemists |
| 21112 | Analytical Chemists |
| 21120 | Biological Scientists and Biochemists |
| 21121 | Biochemists, Medical Scientists |
| 21122 | Biologists |
| 21123 | Bacteriologists, Microbiologists etc. |
| 21124 | Botanists |
| 21125 | Pathologists |
| 21126 | Agricultural Scientists |
| 21127 | Physiologists |
| 21130 | Physicists, Geologists and Meteorologists |
| 21131 | Physicists |
| 21132 | Geophysicists |
| 21133 | Geologists, Mineralogists etc. |
| 21134 | Meteorologists |
| 21135 | Astronomers |
| 21136 | Mathematicians |
| 21210 | Civil Engineers |
| 21211 | Water, Sanitation, Drainage and Public Health Engineers |
| 21212 | Mining, Quarrying and Drilling Engineers |
| 21213 | Construction Engineers |
| 21220 | Mechanical Engineers |
| 21221 | Aeronautical Engineers |
| 21222 | Automobile Engineers |
| 21223 | Marine Engineers |
| 21224 | Plant and Maintenance Engineers |
| 21230 | Electrical Engineers |
| 21231 | Electricity Generation and Supply Engineers |
| 21232 | Telecommunications Engineers |
| 21240 | Electronic Engineers |
| 21241 | Broadcasting Engineers |
| 21242 | Avionics, Radar and Communications Engineers |
| 21250 | Chemical Engineers |
| 21260 | Design and Development Engineers |
| 21270 | Production and Process Engineers |
| 21280 | Planning and Quality Control Engineers |
| 21281 | Planning Engineers |
| 21282 | Quality Control Engineers |
| 21290 | Engineering Professionals Nec |
| 21291 | Metallurgists and Material Scientists |
| 21292 | Patents Examiners, Agents and Officers |
| 21293 | Heating and Ventilating Engineers |
| 21294 | Food and Drink Technologists (including brewers) |
| 21295 | Acoustic Engineers |
| 21310 | IT Strategy and Planning Professionals |
| 21311 | IT Consultants and Planners |
| 21312 | Telecommunications Consultants and Planners |
| 21320 | Software Professionals |
| 21321 | Software Designers and Engineers |
| 21322 | Computer Analysts and Programmers |
| 21323 | Network/Systems Designers and Engineers |
| 21324 | Web Developers and Producers |

**Supplementary Figure 1**. Flowchart for the study design


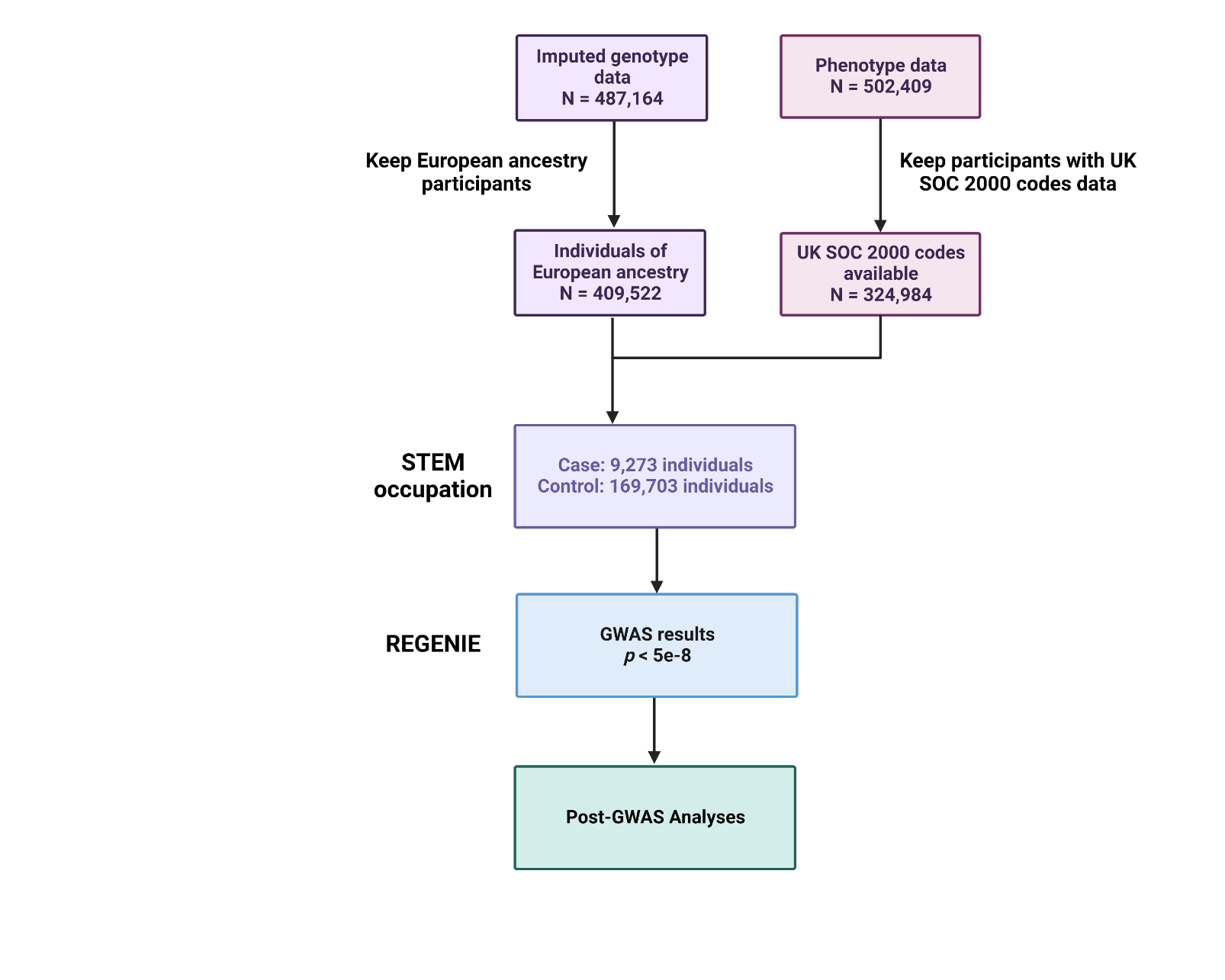


**Supplementary Figure 2**. Associations between an increase of 0.1 in average STEM polygenic score and regional economic performance by local authorities based on birthplace

(a) GDP (b) VAT


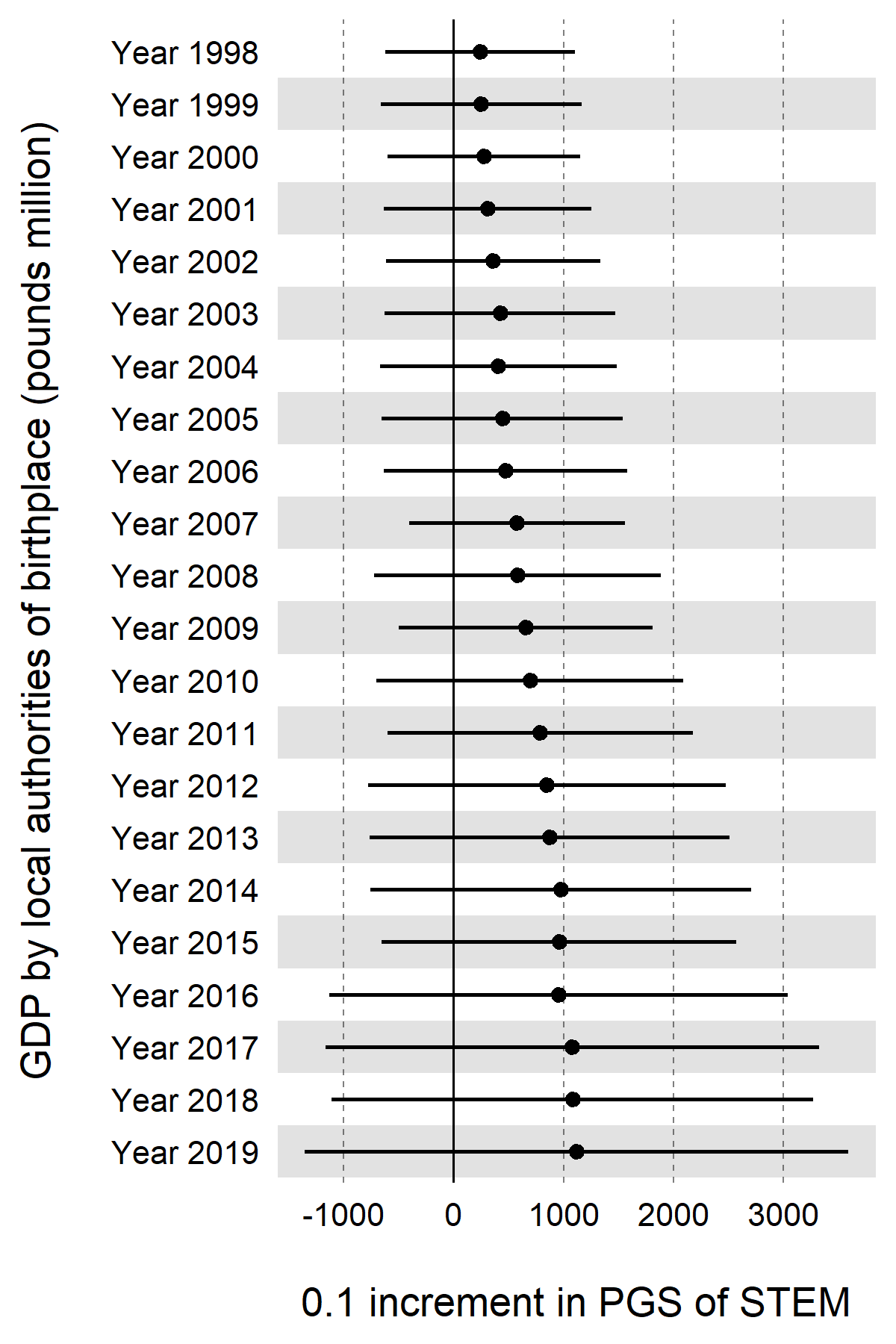

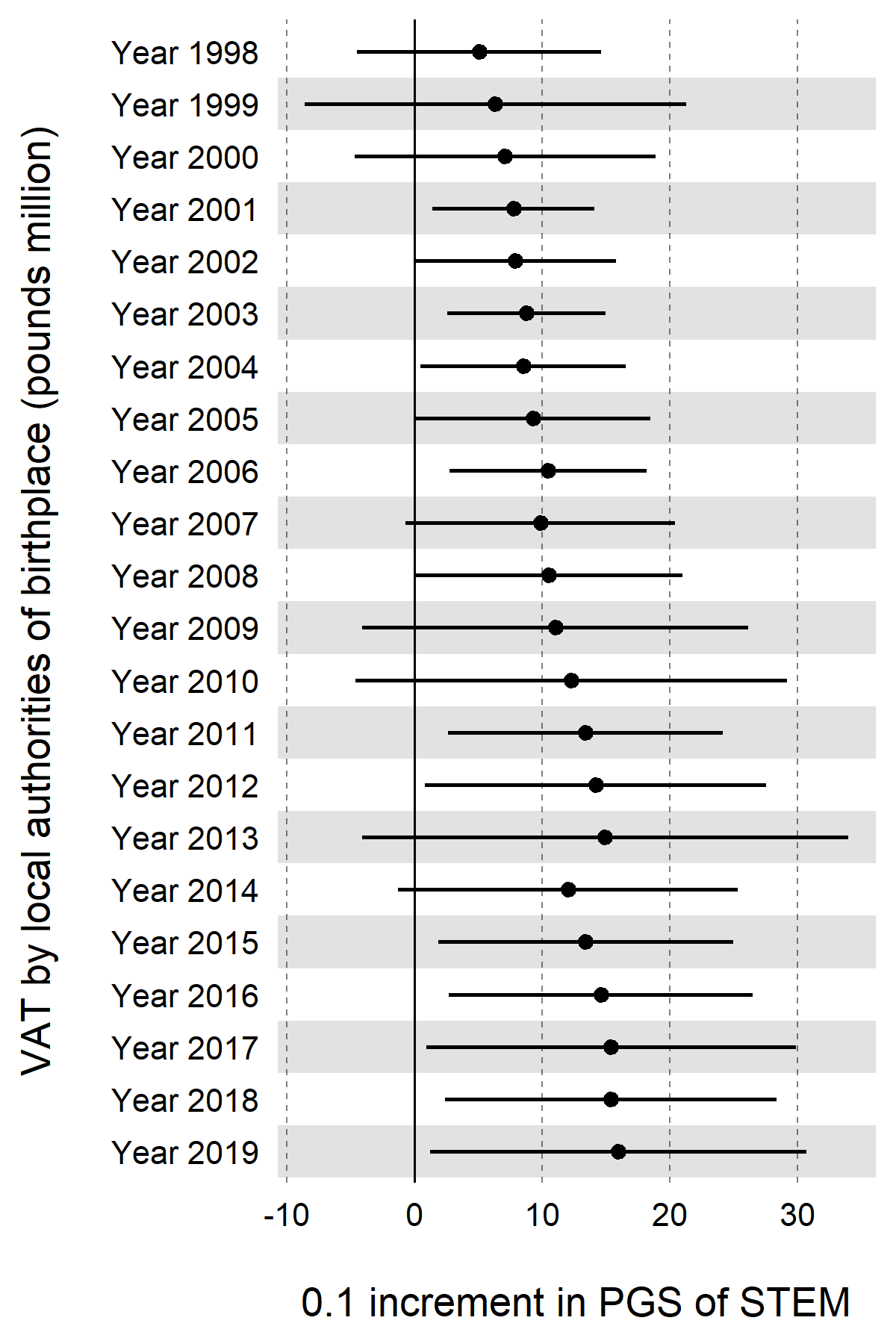


(c) Business counts


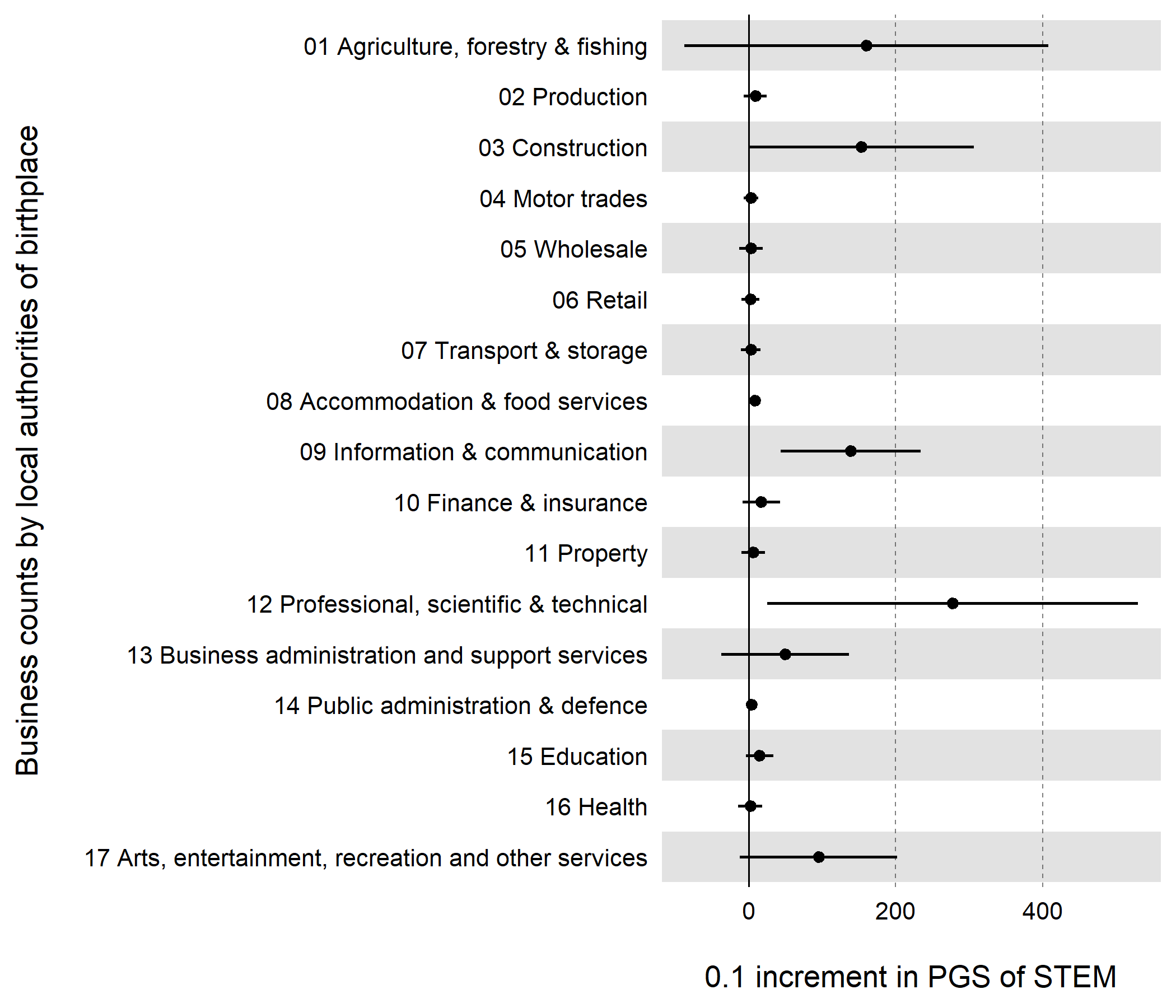


(d) Business employments


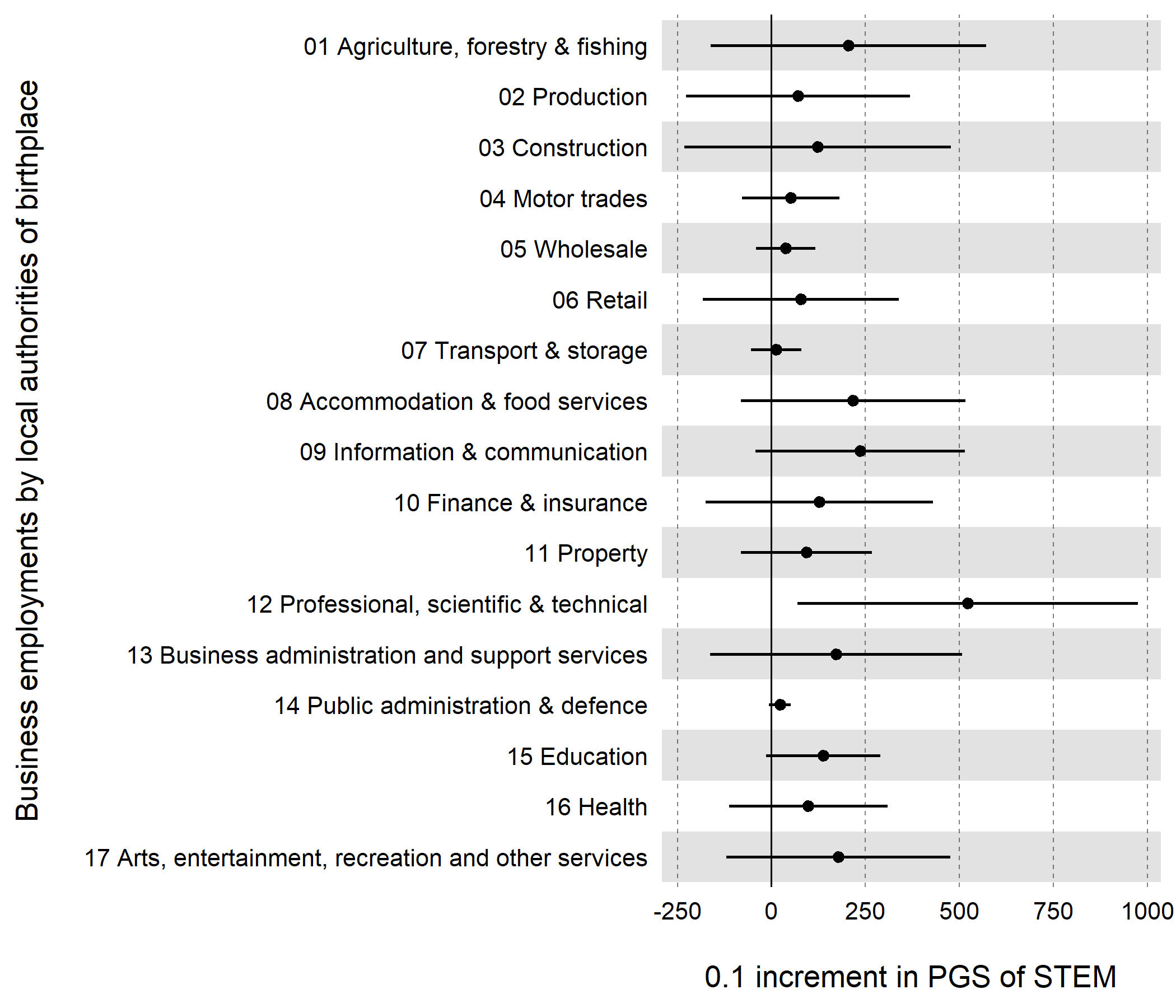

Supplement: Supplementary file 1 — Additional file 1. Supplemental tables and figures. [file 40246_2023_488_MOESM1_ESM.docx]
